# Sequential induction of three recombination directionality factors directs assembly of tripartite integrative and conjugative elements

**DOI:** 10.1371/journal.pgen.1007292

**Published:** 2018-03-22

**Authors:** Timothy L. Haskett, Jason J. Terpolilli, Vinoy K. Ramachandran, Callum J. Verdonk, Phillip S. Poole, Graham W. O’Hara, Joshua P. Ramsay

**Affiliations:** 1 Centre for Rhizobium Studies, School of Veterinary and Life Sciences, Murdoch University, Perth, WA, Australia; 2 Department of Plant Sciences, University of Oxford, Oxford, United Kingdom; 3 School of Pharmacy and Biomedical Sciences and the Curtin Health Innovation Research Institute, Curtin University, Perth, WA, Australia; Swiss Federal Institute of Technology Lausanne (EPFL), SWITZERLAND

## Abstract

Tripartite integrative and conjugative elements (ICE^3^) are a novel form of ICE that exist as three separate DNA regions integrated within the genomes of *Mesorhizobium* spp. Prior to conjugative transfer the three ICE^3^ regions of *M*. *ciceri* WSM1271 ICE*Mc*Sym^1271^ combine and excise to form a single circular element. This assembly requires three coordinated recombination events involving three site-specific recombinases IntS, IntG and IntM. Here, we demonstrate that three excisionases–or recombination directionality factors—RdfS, RdfG and RdfM are required for ICE^3^ excision. Transcriptome sequencing revealed that expression of ICE^3^ transfer and conjugation genes was induced by quorum sensing. Quorum sensing activated expression of *rdfS*, and in turn RdfS stimulated transcription of both *rdfG* and *rdfM*. Therefore, RdfS acts as a “master controller” of ICE^3^ assembly and excision. The dependence of all three excisive reactions on RdfS ensures that ICE^3^ excision occurs via a stepwise sequence of recombination events that avoids splitting the chromosome into a non-viable configuration. These discoveries expose a surprisingly simple control system guiding molecular assembly of these novel and complex mobile genetic elements and highlight the diverse and critical functions of excisionase proteins in control of horizontal gene transfer.

## Author summary

Bacteria evolve and adapt quickly through the horizontal transfer of DNA. A major mechanism facilitating this transfer is conjugation. Conjugative DNA elements that integrate into the chromosome are termed ‘Integrative and Conjugative Elements’ (ICE). We recently discovered a unique form of ICE that undergoes a complex series of recombination events with the host chromosome to split itself into three separate parts. This tripartite ICE must also precisely order its recombination when leaving the current host to avoid splitting the host chromosome and the ICE into non-viable parts. In this work, we show that the tripartite ICEs use chemical cell-cell communication to stimulate recombination and that recombination events are specifically ordered through cascaded transcriptional activation of small DNA-binding proteins called recombination directionality factors. Despite the inherent complexity of tripartite ICEs this work exposes a surprisingly simple system to stimulate their precise and ordered molecular assembly prior to horizontal transfer.

## Introduction

Bacterial genome evolution proceeds at a rapid pace largely due to the sharing of genetic material [1]. This gene exchange is often facilitated by mobile genetic elements (MGEs) such as plasmids, bacteriophage and other chromosomally-integrating elements [2, 3]. MGEs have evolved sophisticated mechanisms to maintain themselves in their host while opportunistically infecting neighbouring organisms, maximising their dissemination through both vertical and horizontal modes of descent [4]. MGEs frequently harbour genes conferring selective benefit to hosts such as virulence, metabolism, symbiosis and antimicrobial-resistance determinants [5–10]. The rapid progress in genome sequencing this century has revealed the ubiquity of MGEs in microbial genomes and specifically, the abundance of MGE-encoded conjugation systems highlights conjugation as a major mechanism of horizontal transmission [11]. It has also become increasingly apparent that ‘non-conjugative’ plasmids and chromosomally-integrating elements may exploit conjugation systems encoded by other MGEs for their own transfer [12–14]. The bacterial mobilome can therefore be viewed as a DNA ecosystem where MGEs compete for an environmental niche defined by the hosts in which they can infect and persist.

Integrative and conjugative elements (ICEs) are the most recently defined MGE, but are probably the most abundant conjugative elements in bacteria [11]. Unlike plasmids, ICEs integrate within their host’s chromosome, negating a strict requirement for full-time extrachromosomal replication systems [15, 16]. Once stimulated to transfer, ICEs excise from the chromosome to form a circular episome capable of conjugation. Rolling-circle replication is an essential part of most conjugation systems so most ICEs likely have the capacity to replicate via this mechanism once excised [17]. Cells carrying an excised ICE can persist in this transfer-competent state and potentially donate ICEs to multiple recipients. Understanding the triggers for ICE transfer requires knowledge of regulatory cues stimulating ICE excision.

Symbiosis ICEs of *Mesorhizobium* spp. are a diverse family of large (~0.5-Mb) ICEs capable of converting non-symbiotic mesorhizobia into symbionts of plant legume species [8, 18–22]. The symbiosis ICE of *M*. *loti* R7A, ICE*Ml*Sym^R7A^, is a 502-kb ICE encoding genes enabling symbiosis with *Lotus* spp. [8, 18, 21, 23]. Integration of ICE*Ml*Sym^R7A^ into mesorhizobial chromosomes is facilitated by the tyrosine recombinase (integrase) IntS [23]. The IntS attachment site *attP*_*S*_ (the subscript denotes the integrase associated with the *att* site) located on the excised circular ICE*Ml*Sym^R7A^ contains a 17-bp DNA sequence identical to the 3’-end of the sole *phe*-tRNA gene (*attB*_*S*_), which is the target for IntS-mediated recombination. Recombination between *attP*_*S*_ and *attB*_*S*_ produces the hybrid sites *attL*_*S*_ and *attR*_*S*_, which flank the integrated ICE*Ml*Sym^R7A^ and together form a direct 17-bp repeat demarcating the ICE*Ml*Sym^R7A^ insertion site [18, 23].

Integrase-mediated recombination can be modulated by additional protein factors that alter the integrase-DNA complex and favoured direction of recombination [24, 25]. Recombination directionality factors (RDFs, or excisionases) are generally small winged-helix-turn-helix domain DNA-binding proteins that bend DNA within integrase *att* sites [25]. Excision of ICE*Ml*Sym^R7A^ requires the RDF RdfS (S1 Fig). Overexpression of *rdfS* cures ICE*Ml*Sym^R7A^ from *M*. *loti* R7A cells producing the non-symbiotic derivative R7ANS [23]. A synthetic non-replicative mini-ICE carrying only *attP*_*S*_ and *intS* is able to integrate into the *attB*_*S*_ site of R7ANS, confirming IntS is the only ICE*Ml*Sym^R7A^ protein required for integration. Subsequent introduction of a plasmid constitutively expressing *rdfS* stimulates loss of the integrated mini-ICE from R7ANS [23], suggesting that like other excisionases, RdfS probably binds the IntS attachment sites to stimulate IntS-catalysed formation of *attP*_*S*_ and *attB*_*S*._

**Fig 1:**
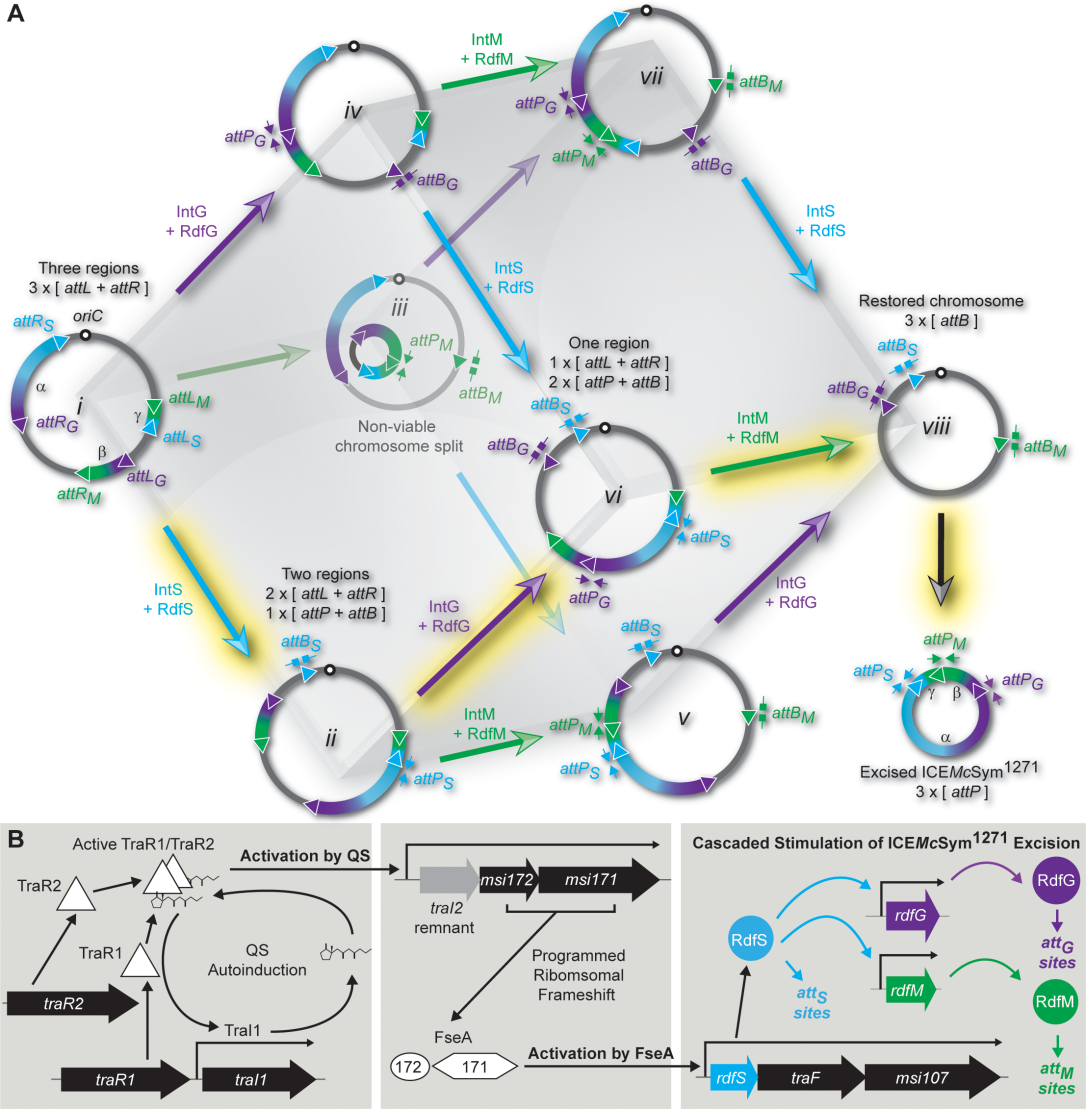
ICE*Mc*Sym^1271^ assembly, excision and regulation. (A) Schematic of the possible ICE*Mc*Sym^1271^ recombination states and recombination reactions leading to formation of excised ICE*Mc*Sym^1271^ assembled from regions α, β and γ. The recombination targets *attP*, *attB*, *attL* and *attR* (triangles) and recombination reactions (large arrows) are color-coded for each integrase: cyan for IntS; magenta for IntG; green for IntM. Primers for qPCR measurement of recombination are indicated as block-headed arrows for *attB* sites and triangle-headed arrows for *attP* sites (see Materials and Methods and S1 Table for details). Data presented here support a model where RDF-stimulated excisive reactions occur in the order IntS > IntG > IntM (highlighted in yellow) to produce excised ICE*Mc*Sym^1271^. (B) The regulatory model of quorum-sensing mediated stimulation of ICE*Mc*Sym^1271^ assembly and excision. TraR1 and TraR2 bind AHLs produced by TraI1. TraR1/2-AHL complex(es) activate transcription from the *traI1* and *traI2* promoters. *traI2-msi172-msi171* expression leads to production of FseA and transcriptional activation of the *rdfS* operon. RdfS stimulates excisive IntS-mediated recombination and promotes expression of RdfG and RdfM. RdfG stimulates the excisive IntG-mediated reaction and RdfM stimulates excisive IntM-mediated recombination and excision.

Recently we identified a new form of ICE, termed a tripartite ICE (ICE^3^), composed of three separated chromosomal regions of DNA α, β and γ [19, 26]. Three site-specific recombination reactions assemble these ICE^3^ regions into a single circular entity prior to conjugation. The ICE^3^ of *M*. *ciceri* WSM1271 (ICE*Mc*Sym^1271^) carries homologues of *rdfS*, *intS* and all genes identified as being required for horizontal transfer of ICE*Ml*Sym^R7A^. However, ICE*Mc*Sym^1271^ carries two additional tyrosine recombinases IntG and IntM, two additional predicted excisionases RdfG and RdfM and two additional sets of attachment sites *attL*_*G*,_ *attR*_*G*_, *attP*_*G*_, *attB*_*G*_, and *attL*_*M*,_ *attR*_*M*_. *attP*_*M*_, *attB*_*M*_ (Fig 1A) [19]. Using a synthetic non-replicative mini-ICE^3^ element containing all three *attP* sites derived from ICE*Mc*Sym^1271^, IntS, IntG and IntM were demonstrated to mediate chromosomal integration and subsequent dispersal of this mini-ICE^3^ into the separate regions α, β and γ [19]. We additionally identified numerous putative tripartite ICEs in diverse symbiotic mesorhizobia, each carrying unique genetic cargo in each ICE^3^ region. We propose that the tripartite integration pattern serves to stabilize the ICE in the host and protect it from potential destabilisation by competing ICEs and other integrative elements [26].

The increased complexity introduced by the three separate recombination reactions required for ICE*Mc*Sym^1271^ integration and excision allows for the potential formation of eight distinct chromosomal recombination states [19]. The arrival at any particular state depends on the prior order and direction of the recombination reactions catalysed by IntS, IntG and IntM. Not all eight states can be reconstructed using the mini-ICE^3^, suggesting some states are non-viable. Specifically, our model indicates that if the first excisive reaction is catalysed by IntM, i.e. *attL*_*M*_ + *attR*_*M*_ > *attP*_*M*_ + *attB*_*M*_, then the chromosome is split into two parts, one part lacking the likely essential *phe* and *his*-tRNA genes and the other part an origin-of-replication (Fig 1A) (ICE^3^ recombination reactions producing *attP* + *attB* do not necessarily result in ICE^3^ excision *per se*, but for simplicity will be referred as ‘excisive’). Quantitative PCR (qPCR) assays measuring IntM-mediated formation of *attP*_*M*_ + *attB*_*M*_ indicate the excisive IntM reaction occurs at the lowest frequency of the three integrase-mediated reactions [19], suggesting evolved regulatory control mechanisms might prevent IntM-mediated excisive recombination occurring before other reactions, precluding formation of the non-viable chromosome state.

In this work, we show that the three excisive reactions of ICE*Mc*Sym^1271^ are dependent on three distinct RDFs, RdfG, RdfM, and RdfS. ICE*Mc*Sym^1271^ excision and transfer is stimulated by quorum sensing (QS). RNA sequencing (RNAseq) revealed QS activation results in activation of *rdfS* expression (Fig 1B). Surprisingly, all three *attL* + *attR* > *attP* + *attB* reactions were dependent on *rdfS* and we demonstrated that this is because RdfS activates expression from the *rdfG* and *rdfM* promoters. Therefore, the ordered assembly and excision of ICE*Mc*Sym^1271^ is accomplished through a cascade of transcriptional activation initiated by QS and finalised by RdfS, ensuring RdfS is always the first excisionase translated and that IntS-catalysed excisive recombination occurs ahead of the IntG and IntM-catalysed reactions.

## Results

### RdfG and RdfM are required for excisive IntG and IntM-mediated recombination

**Fig 2:**
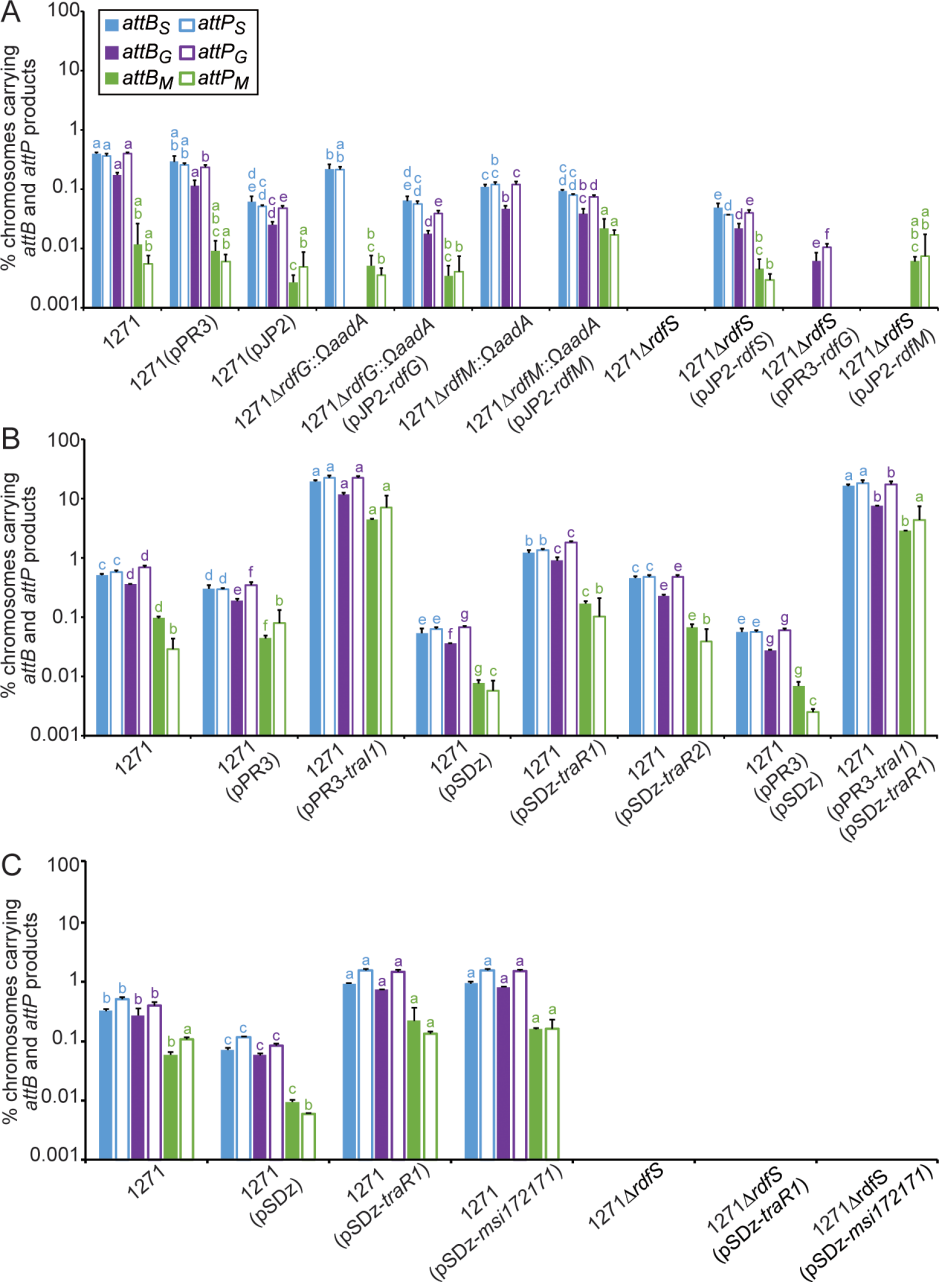
qPCR measurement of excisive ICE*Mc*Sym^1271^ recombination. Measurements represent the mean percentage of WSM1271 chromosomes in stationary-phase cultures harbouring each excisive Int-mediated recombination product (*attB*_*S*_, *attP*_*S*_, *attB*_*G*_, *attP*_*G*_, *attP*_*M*_, *and attP*_*M*_) determined by qPCR [19]. Where appropriate, plasmids carried by WSM1271 (here abbreviated as 1271) are listed in brackets after the strain name (see Table 3 for a description of plasmids). Values for each of the assay types *attB*_*S*_, *attP*_*S*_, *attB*_*G*_, *attP*_*G*_, *attP*_*M*_, and *attP*_*M*_ site were individually compared between strains within the same panel (panel A, B, or C) using ANOVA and Fisher’s LSD test controlling for type I error using the Bonferroni adjustment. Groups of values from the same assay type and in the same panel that are not significantly different from each other have the same letter (a, b, c, d, e, f or g) indicated above. Expression from the IPTG inducible promoter of pSDz constructs were not induced with IPTG as they exhibit leaky expression without induction in TY medium used for assays. (A) Involvement of *rdfG* and *rdfM* in excisive recombination. (B) Quorum-sensing induction of excisive recombination. (C) Involvement of *rdfS* in excisive recombination.

Integration (formation of *attL* + *attR* from *attP* + *attB*) and excision (formation of *attP* + *attB* from *attL* + *attR*) of ICE*Ml*Sym^R7A^ are catalysed by the integrase IntS, however, integration is favoured in the absence of RdfS. Overexpression of *rdfS* in R7A stimulates the excision reaction and results in loss of ICE*Ml*Sym^R7A^ from the cell [23]. In contrast, the tripartite ICE*Mc*Sym^1271^ of WSM1271 requires the concerted action of three integrases IntG, IntM, and IntS to direct integration and excision [19]. In addition to a homologue of *rdfS*, two other putative excisionase genes *rdfG* and *rdfM* are present on ICE*Mc*Sym^1271^ [19, 26]. *rdfG* is oriented convergently with *intG* on ICE*Mc*Sym^1271^ region β and *rdfM* is encoded directly upstream of *intM* on ICE*Mc*Sym^1271^ region γ. Like RdfS, RdfG (Mesci_2550) and RdfM (Mesci_2345) are MerR superfamily proteins with a predicted winged-helix-turn-helix secondary structure (S1 Fig). To investigate potential roles of *rdfG* and *rdfM* we replaced each gene with an *ΩaadA* cassette producing strains 1271Δ*rdfG*::Ω*aadA* and 1271Δ*rdfM*::Ω*aadA*, respectively, and using our previously validated qPCR assay [19], measured the abundance of each the three pairs of *attP* and *attB* sites formed following each of the three excisive reactions. In wild-type WSM1271, *attP*_*G*_ + *attB*_G_ and *attP*_S_ + *attB*_*S*_ sites were detected at a frequency of 0.1–1% per chromosome and *attP*_M_ + *attB*_*M*_ sites were detected at 0.01–0.1% (Fig 2A). In contrast, *attP*_*G*_ + *attB*_G_ sites were undetectable in 1271Δ*rdfG*::Ω*aadA* and *attP*_*M*_ + *attB*_M_ sites were undetectable in 1271Δ*rdfM*::Ω*aadA*. The abundance of the two remaining pairs of *attP* + *attB* sites in each of these mutant strains was similar to that of WSM1271. Complementation of 1271Δ*rdfG*::Ω*aadA* with a cloned copy of *rdfG* and its native promoter partially restored *attP*_*G*_ + *attB*_*G*_ formation and complementation of 1271Δ*rdfM*::Ω*aadA* with a cloned copy of *rdfM* and its native promoter restored *attP*_*M*_ + *attB*_*M*_ production. These experiments therefore confirmed the roles of RdfG and RdfM in excisive IntG and IntM reactions, respectively.

### Quorum sensing stimulates all three excisive Int-mediated recombination reactions

All three pairs of ICE*Mc*Sym^1271^ *attP* and *attB* products are most abundant in stationary-phase cultures and the α region of ICE*Mc*Sym^1271^ carries a subset of genes homologous to those involved in quorum-sensing (QS) regulation of ICE*Ml*Sym^R7A^ excision and conjugative transfer in R7A [19, 23, 27]. These QS genes include a homologue of the ICE*Ml*Sym^R7A^ *N-*acyl-homoserine lactone (AHL)-synthase gene *traI1* (Mesci_5572) and a homologue of the AHL-dependent transcriptional regulator *traR* (Mesci_5573), here named *traR1*. A second *traR* homologue (Mesci_5676), here named *traR2*, is present on ICE*Mc*Sym^1271^-α adjacent to a homologue of the QS antiactivation gene *qseM* [28]. Therefore, we suspected that excision and conjugative transfer of ICE*Mc*Sym^1271^ was under QS control.

To confirm ICE*Mc*Sym^1271^ *traI1* encoded a functional AHL synthase, *traI1* was cloned into pPR3 downstream of the *nptII* promoter. The resulting plasmid pPR3-*traI1* was introduced into *E*. *coli* DH5α and *M*. *loti* R7ANS and the resulting strains were screened for AHL production using the *Chromobacterium violaceum* CV026 AHL bioassay [29]. CV026 violacein production was induced by *E*. *coli* and *M*. *loti* strains carrying the pPR3-*traI1* vector (S2 Fig), but not by strains carrying the vector-only control pPR3, indicating that *traI1* produced C_4_-C_8_ AHLs in both backgrounds. In *M*. *loti* R7A, AHL-activated TraR stimulates transcription of *traI1* completing a positive feedback loop of regulation [27]. To confirm TraR1 and TraR2 activated expression from the *traI1* promoter of ICE*Mc*Sym^1271^ (*P*_*traI1*_), *traR1* and *traR2* were cloned downstream of the *lac* promoter on a derivative of pSDz also carrying *P*_*traI1*_ upstream of the *lacZ* gene. The resulting constructs were mobilized into R7ANS additionally carrying either pPR3-*traI1* or pPR3. β-galactosidase assays of the resulting strains revealed that both *traR1* and *traR2* partially induced expression from *P*_*traI1*_ in the absence of *traI1* and other ICE-encoded genes_,_ however, maximum expression from this promoter was only achieved in the presence of *traI1* (S3 Fig).

*traI1*, *traR1* and *traR2* were next each individually overexpressed in WSM1271 on plasmids and ICE^3^ excision was measured by qPCR (Fig 2B). Constitutive expression of *traI1* from the *nptII* promoter stimulated a 10-100-fold increase in abundance all three *attP* + *attB* sites relative to vector-only controls. Non-induced *lac* promoter*-*driven expression of *traR1* or *traR2* only stimulated a modest increase in *att* site abundance relative to WSM1271, however, unexplainedly the vector-only control exhibited ~10-fold reduced excision frequencies, so relative to this background overexpression of the *traR1/2* genes each induced a 10-100-fold increase for all *attP* + *attB* sites. Overexpression of *traI1* and *traR1* in the same background stimulated ~1000-fold increase in abundance all three *attP* + *attB* sites relative to the vector-only control strain. To investigate effects of the QS genes on conjugative transfer, strains overexpressing *traR1*, *traR2*, and *traI1* were each used as donors in mating assays where *M*. *loti* R7ANS carrying pPR3 or pFAJ1708 was the recipient (Table 1). The pattern of fold-changes in conjugation frequencies for each donor strain largely mirrored excision frequency changes observed in qPCR assays (Fig 2B) confirming that *traI1*, *traR1* and *traR2* also stimulated conjugative transfer.

**Table 1:** Quorum-sensing induced ICE*Mc*Sym^1271^ conjugative transfer.

^a^^b^Donor	Recipient	Exconjugants (per donor)	Standard deviation	^c^Fold-change
WSM1271	R7ANS(pPR3)	8.02 x 10^−8^	1.82 x 10^−8^	-
WSM1271(pSDz)	R7ANS(pPR3)	2.22 x 10^−8^	9.12 x 10^−9^	-
WSM1271(pSDz-*traR1*)	R7ANS(pPR3)	4.69 x 10^−7^	1.11 x 10^−7^	21.14
WSM1271(pSDz*-traR2*)	R7ANS(pPR3)	5.97 x 10^−7^	1.66 x 10^−7^	26.90
WSM1271(pSDz-*msi172171*)	R7ANS(pPR3)	8.49 x 10^−7^	8.23 x 10^−8^	38.30
WSM1271	R7ANS(pFAJ1708)	8.35 x 10^−8^	4.87 x 10^−8^	-
WSM1271(pPR3)	R7ANS(pFAJ1708)	8.74 x 10^−8^	3.89 x 10^−8^	-
WSM1271(pPR3-*traI1*)	R7ANS(pFAJ1708)	1.04 x 10^−5^	1.50 x 10^−6^	119.06

^a^ Where appropriate, plasmids carried by WSM1271 are listed in brackets after the strain name (see Table 3 for a description of plasmids).

^b^ Expression from the IPTG inducible promoter of pSDz constructs were not induced with IPTG as they exhibit leaky expression without induction in TY medium used for assays.

^c^ Fold-change is relative to control strains carrying the appropriate pPR3 or pSDz parent vector.

### Dissection of quorum sensing-induced ICE^3^ excision using RNA deep sequencing

QS-induced excision and conjugative transfer of ICE*Ml*Sym^R7A^ is dependent on the transcriptional activation of *rdfS* [30]. In *M*. *loti* R7A, AHL-activated TraR stimulates transcription from ‘*tra-*box’ promoters centred 69-bp upstream of *traI1* and 67-bp upstream of the *traI2-msi172-msi171* operon. A programmed ribosomal frameshift site encoded in the 3’ end of *msi172* facilitates translational fusion of Msi172 and Msi171, producing FseA, a regulator essential for transcription from the *rdfS* promoter [27, 31, 32]. Homologues of *msi172-msi171* and *rdfS* are also located on the α region of ICE*Mc*Sym^1271^ [19], therefore it seemed likely these genes also stimulated ICE*Mc*Sym^1271^ excision. Transcriptome sequencing (RNAseq) was carried out for a QS-induced (QS+) strain carrying plasmid-borne copies of *traI1* and *traR1* and an uninduced strain (QS-) carrying the appropriate empty vectors. Overall, 187 significantly differentially expressed genes (adjusted *P*-value < 0.05) were identified (S1 Dataset) and although ICE*Mc*Sym^1271^ comprised only ~7.6% of the chromosome, 29 (15.5%) of the differentially expressed genes were located on ICE*Mc*Sym^1271^. Genes likely involved in activation of excision and conjugation including *rdfS*, *rlxS* and the type-IV conjugative pilus gene cluster *msi031-trbBCDEJLFGI-msi021* were all significantly induced (Table 2).

**Table 2:** Quorum-sensing induced/repressed ICE*Mc*Sym^1271^-encoded genes.

Gene	Locus ID	^a^Fold-change	Standard error
**Region-α**			
*rdfS*	Mesci_5530	19.74	1.20
*traF*	Mesci_5529	29.21	1.20
*msi107*	Mesci_5528	41.10	1.19
*rlxS*	Mesci_5527	58.14	1.17
*PtraI1*	-	121.45	1.16
*P traI2*	-	37.54	1.18
*traI2*	-	141.41	1.16
*msi172*	-	61.71	1.18
*msi171*	-	156.99	1.16
*msi021*	Mesci_5513	8.28	1.19
*trbI*	Mesci_5514	10.58	1.17
*trbG*	Mesci_5515	18.07	1.19
*trbF*	Mesci_5516	14.48	1.19
*trbL*	Mesci_5517	19.35	1.19
*trbJ*	Mesci_5518	42.31	1.18
*trbE*	Mesci_5519	64.16	1.17
*trbD*	Mesci_5520	14.43	1.20
*trbC*	Mesci_5521	9.71	1.20
*trbB*	Mesci_5522	5.39	1.21
*msi031*	Mesci_5523	13.88	1.20
*traG*	Mesci_5524	2.75	1.16
*queD*	Mesci_5560	-2.35	0.83
*queC*	Mesci_5561	-2.29	0.82
*queB*	Mesci_5562	-2.34	0.83
hypothetical	Mesci_5526	1.90	1.18
**Region-β**			
*cbb3*-type COx (SI)	Mesci_5510	1.92	1.16
Nicotinate biosynthesis protein	Mesci_5579	-1.85	0.83
*rdfG*	Mesci_2550	2.46	1.18
Hypothetical	Mesci_2555	2.03	1.19
**Region-γ**			
*intS*	Mesci_2349	2.85	1.15

^a^ Differentially expressed genes (adjusted two-sided *P-*value of < 0.05) were identified using the DESeq2 package [33]. Since introduced plasmids carried copies of the *traI1* and *traR* ORFs (not including promoter regions), reads mapping to these sequences were of an ambiguous origin and were therefore filtered and removed prior to mapping reads. Differential expression analysis of the *traI1* and *traI2* untranslated mRNA promoter regions, *P*_*traI1*_ and *P*_*traI2*_, was carried out prior to filtering–as these reads were able to be distinguished from plasmid-borne mRNAs. Reads mapping to the plasmid backbones and rRNA genes were removed prior to mapping reads for both analyses.

An alignment of the *P*_*traI1*_ regions from ICE*Ml*Sym^R7A^ and ICE*Mc*Sym^1271^ revealed a *tra-*box sequence centred 69bp upstream of the ICE*Mc*Sym^1271^ *traI1* start codon (S4A Fig). The reads mapping to the *traI1* coding sequence were filtered from our RNAseq libraries prior to differential expression analyses (Table 2) because they were also present on the introduced plasmid, however, a secondary comparison of the unfiltered RNAseq reads mapping to the *P*_*traI1*_ region in our QS+ relative to the QS- WSM1271 cells revealed a sharp 121-fold increase in mapped reads beginning 44bp downstream from *tra-*box centre and 26bp upstream of the *traI1* start codon (Table 2 & S4A Fig).

**Fig 3:**
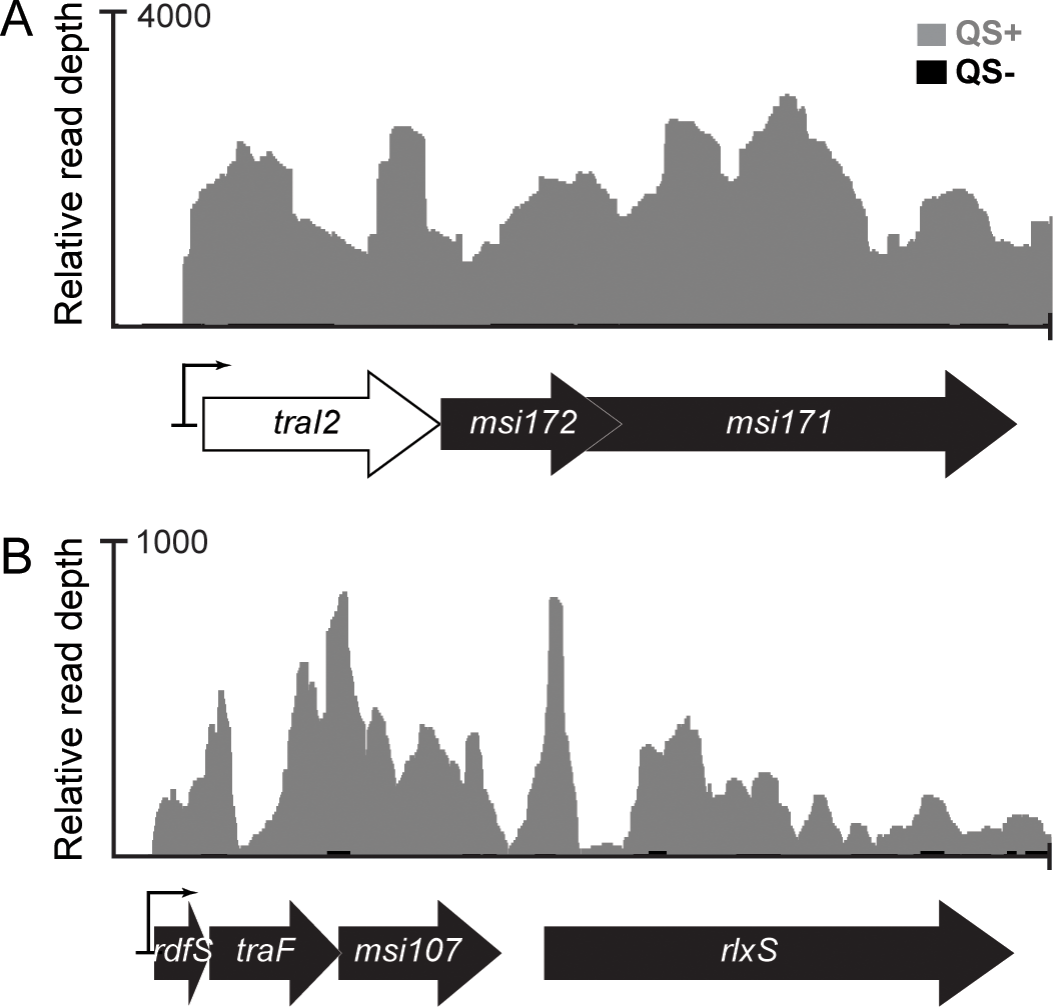
Quorum sensing activation of ICE*Mc*Sym^1271^ promoters. Overlayed relative read coverage (or sequencing depth) plots represent standardised values for the mean number of reads mapped to the positive strand of the regions shown in this figure from the three unfiltered QS+ (grey) and QS- (black) transcriptome libraries of WSM1271. QS+ strains were induced for QS by overexpressing both *traI1* and *traR1* from the plasmids pPR3-*traI1* and pSDz*-traR1*, respectively, whereas the QS- control strains carried the parent vectors pPR3 and pSDz. The mean read depth for the (A) *traI2-msi172-msi171*, and (B) *rdfS-traF-msi107* and *rlxS* regions of ICE*Mc*Sym^1271^ in QS- transcriptome libraries were almost non-existent relative to that of the QS+ strain (See S1 Dataset for a full list of TPM values from the filtered reads). A magnified view of reads mapping to the promoter region and the DNA sequence is shown in S4 Fig. These plots were produced using Integrated Genome Browser [73].

Homologues of *msi172* and *msi171* are present on ICE*Mc*Sym^1271^ (Fig 3A) [19] but our initial interrogations did not identify an ICE*Ml*Sym^R7A^ *traI2* homologue positioned upstream of these genes. *traI2* of ICE*Ml*Sym^R7A^ appears to encode an AHLsynthase paralogous with TraI1, however, mutation of *traI2* has no effect on ICE*Ml*Sym^R7A^ excision and no identifiable AHL products are produced by TraI2 [27]. Further inspection of the ICE*Mc*Sym^1271^ *msi172-msi171* region revealed the presence of a potential *tra-*box sequence centred 398bp upstream of the *msi172* start codon (S4A Fig). A nucleotide alignment with the corresponding ICE*Ml*Sym^R7A^ region revealed this *tra*-box was also centred 66bp upstream of an internally-truncated *traI2* gene remnant (S4A Fig). This *traI2* pseudogene overlapped the start codon of *msi172* as does *traI2* on ICE*Ml*Sym^R7A^ (Fig 3A). Interestingly, inspection of *traI2-msi172* regions in *M*. *loti* USDA 3471 and *M*. *ciceri* strains WSM4083, WSM1497, and WSM1284 revealed a similar situation; the *traI2* gene in each case was present as a potential protein-coding pseudogene upstream of *msi172* and overlapping the *msi172* start codon (S5A and S5B Fig). Therefore, although *traI2* has likely become a pseudogene on ICE*Mc*Sym^1271^ and other symbiosis ICE/ICE^3^s, the transcriptional coupling of the *tra-*box and translational coupling of the TraI2 and Msi172 coding sequences has been maintained. In our RNAseq experiments, *traI2*, *msi172* and *msi171* reads were increased ~60-160-fold in QS+ cells (Table 2). A sharp increase in relative read depth was observed at the *traI2* promoter 44bp downstream of the *tra*-box centre and 21bp upstream of the *traI2* start codon (S4B Fig) which spanned the entire *traI2-msi172-msi171* operon (Fig 3A). The likely transcription start site for *traI2* observed from RNAseq reads was consistent with the previously mapped ICE*Ml*Sym^R7A^ *traI2* promoter (S4B Fig) [27]. Interestingly, comparison of the number of unfiltered transcripts mapping to the *traI1* and *traI2* promoter regions revealed that QS-induced expression from the *traI1* promoter (2196.16 ± [SE] 434.70 TPM) is ~3-fold stronger than that of *traI2* (660.88 ± 276.84 TPM) (S4A & S4B Fig). A similar ratio of *traI1*:*traI2* expression is also observed for ICE*Ml*Sym^R7A^ [27].

For ICE*Ml*Sym^R7A^, FseA stimulates expression from an operon containing *rdfS*, *traF* and *msi107* [21, 31] (Fig 3B). The same gene cluster is present on ICE*Mc*Sym^1271^ and the RNAseq read depth for the corresponding ICE*Mc*Sym^1271^ homologues was increased 20-58-fold in QS+ cells (Table 2). A distinct read depth increase was observed 25bp upstream of the *rdfS* start codon corresponding closely with the mapped transcriptional start site for ICE*Ml*Sym^R7A^ *rdfS* (S4C Fig) [31]. In summary, despite several genetic rearrangements, the QS regulon of ICE*Mc*Sym^1271^ appears functionally analogous to that of ICE*Ml*Sym^R7A^ and importantly, QS induces the expression of *msi172*, *msi171* and *rdfS*.

### *rdfS* is required for all three excisive Int-mediated recombination reactions

To explore the involvement of RdfS in ICE^3^ assembly and excision, a markerless deletion in the WSM1271 *rdfS* gene was constructed. As expected, no *attP*_*S*_ or *attB*_*S*_ products were detected in this strain, but interestingly *attP*_*G*_ + *attB*_*G*_ and *attP*_*M*_ + *attB*_*M*_ products were also undetectable (Fig 2A). Introduction of *rdfS* expressed from its native promoter restored *attP* + *attB* production at all three sites, albeit at lower levels than wild-type WSM1271. Plasmid-based overexpression of *traR1* or *msi172-msi171* in the *rdfS* mutant did not induce excision, however, the same plasmids did induce excision and conjugative transfer in the wild-type WSM1271 (Fig 2C and Table 1). Together these data confirmed that the stimulation and coordination of all three excision reactions by QS and *msi172-msi171* is dependent on *rdfS*.

We hypothesized that RdfS was either directly required to stimulate excisive recombination at *att*_*G*_ and *att*_*M*_ sites or that RdfS stimulated *rdfG* and *rdfM* expression. We overexpressed *rdfG* and *rdfM* in the *rdfS* mutant to see if it would restore the formation of *attP*_*G*_ + *attB*_*G*_ and *attP*_*M*_ + *attB*_*M*_ sites, respectively. *rdfG* was cloned downstream of the strong constitutive *nptII* promoter and *rdfM* was cloned downstream of the *lac* promoter. Interestingly, introduction of *lac*-driven *rdfM* resulted in growth arrest even in the absence of IPTG inducer and in the presence of glucose to repress *lac* expression. This was consistent with our model for excision, in which expression of *rdfM* alone splits the chromosome and results in loss of viability. Constitutive expression of *rdfG* in the *rdfS* mutant resulted in the restored detection of *attP*_*G*_ + *attB*_*G*_ products in approximately 0.01% of cells (Fig 2A) while the other two sites remained undetectable. In contrast to *lac-*driven expression, introduction of the cloned copy of *rdfM* downstream of its native promoter restored the production of *attP*_*M*_ + *attB*_*M*_ sites in 0.001–0.01% of cells. Therefore, it was clear that *attP + attB* formation was abolished in the *rdfS* mutant but RdfS was not directly essential for excisive IntG and IntM recombination. The observation that artificially increased levels of *rdfG* or *rdfM* compensated for the loss of *rdfS* implied RdfG and RdfM expression was abolished in the *rdfS* mutant.

### Overexpression of *rdfS* stimulates expression of *rdfG* and *rdfM*

**Fig 4:**
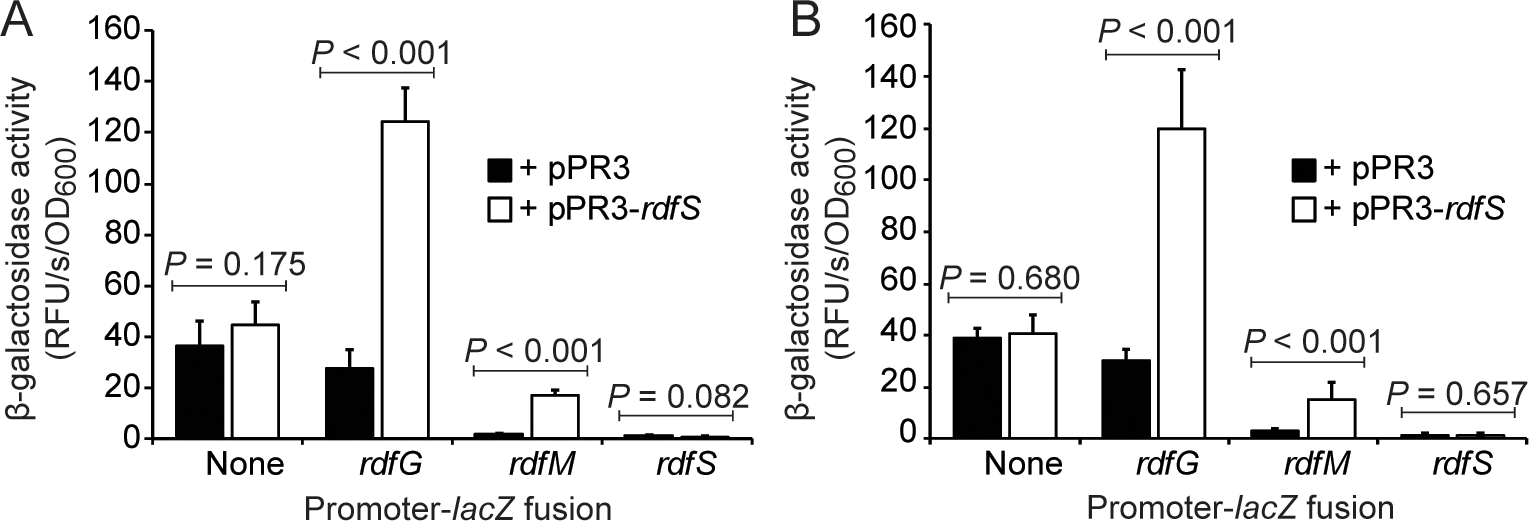
Transcriptional regulation of *rdfG* and *rdfM* by RdfS. β-galactosidase assays [67] were performed for (A) WSM1271 and (B) R7ANS carrying either control vector pPR3 or pPR3-*rdfS* (constitutively expressing *rdfS*) together with one of three RDF promoter-*lacZ* fusion constructs cloned into the pSDz vector. Assays were performed with six biological replicates and mean β-galactosidase activity values (Relative Fluorescent Units/s/OD_600_) were compared by Bonferroni adjusted student’s t-tests. SD is denoted by error bars.

Inspection of RNAseq data revealed *rdfG* mRNA abundance was ~2.5-fold higher in QS+ cells (Table 1). *rdfM* was very weakly expressed in both QS+ and QS- cells and while there was ~2-fold more *rdfM* reads in QS+ cells, this difference was not statistically significant. To clarify the potential role for RdfS in activation of the *rdfG* and *rdfM* promoters, the non-coding regions present upstream of each gene were cloned upstream of the promoterless *lacZ* gene. Plasmid constructs carrying this fusion were introduced into WSM1271 carrying a constitutively expressed copy of *rdfS* (Fig 4A). β-galactosidase expression from the *rdfG* and *rdfM* promoters was induced ~4.5 and ~8-fold respectively in the presence of constitutively expressed *rdfS*. Consistent with RNAseq data, *rdfM* expression was much lower than *rdfG* expression and almost undetectable in the absence of *rdfS*. To discount the possibility that RdfS induced expression indirectly through other factors on ICE*Mc*Sym^1271^, the same set of experiments were repeated using the heterologous *M*. *loti* R7ANS background, which lacks all ICE genes (Fig 4B). These assays produced comparable results to those carried out in WSM1271, supporting the hypothesis that the transcriptional activation of *rdfG* and *rdfM* promoters by RdfS was likely direct.

## Discussion

Excision and circularization is an essential prerequisite for conjugative transfer of ICEs. Integrase proteins of ICEs and temperate phages generally catalyse both the excision and integration reactions, but integrative recombination is generally favoured in the absence of a cognate RDF [25]. Unlike most ICEs that excise following a single Int-mediated recombination, ICE*Mc*Sym^1271^ requires three Int-mediated reactions to excise [19]. Here, we demonstrated that three ICE*Mc*Sym^1271^-encoded RDFs RdfG, RdfM, and RdfS are required for the ICE*Mc*Sym^1271^ excisive IntG, IntM, and IntS-mediated recombination reactions, respectively. We also demonstrated that overexpression of the QS sensors TraR1 and TraR2 or autoinducer synthase TraI1 in WSM1271 simultaneously increased the proportion of cells in a population undergoing all three ICE*Mc*Sym^1271^ excision reactions 10-100-fold. QS significantly induced mRNA abundance for the WSM1271 *traI1*, *traI2-msi172-msi171*_,_ *rdfS*, and *rdfG* genes, as well as those for conjugative pilus formation [23, 27, 31]. In addition to stimulating the ICE*Mc*Sym^1271^ IntS-mediated excisive recombination, RdfS was shown to transcriptionally activate the *rdfG* and *rdfM* genes. Therefore, RdfS acts as the master regulator for ICE*Mc*Sym^1271^ excision.

Our model for the assembly and excision of ICE*Mc*Sym^1271^ indicates that if the first excisive reaction is catalysed by IntM, then the chromosome is split into two inviable parts (Fig 1A). However, transcription of *rdfM* and *rdfG* is dependent on RdfS, and thus excisive IntS-mediated recombination probably occurs prior to that of IntM and IntG in WSM1271 cells induced for ICE*Mc*Sym^1271^ assembly an excision. This hierarchical genetic regulation of the three RDFs has likely evolved to minimise the potential for formation of the non-viable split chromosome configuration following spurious *rdfM* expression. In wild-type WSM1271 or QS-induced WSM1271 cells, the frequency *attP*_*M*_ *+ attB*_*M*_ site formation was also significantly less than either that of *attP*_*G*_ *+ attB*_*G*_ and *attP*_*S*_ *+ attB*_*S*_, as was expression of *rdfM* relative to *rdfG* and *rdfS*. Moreover, introduction of a plasmid-borne copy of *rdfM* under the control of the relatively weak *lacI* promoter on pSacB [34] resulted in arrested growth of 1271Δ*rdfS* cells suggesting that even a low level of RdfM expression in the absence of RdfS and RdfG is deleterious. It is possible that the *rdfM* promoter, in addition to evolving transcriptional dependency on RdfS, has evolved to promote only subtle levels of *rdfM* expression to further reduce the likelihood of the formation of a non-viable chromosomal state. Considering the data, it seems probable that the *in situ* excisive recombination pathway of ICE*Mc*Sym^1271^ follows the sequence IntS > IntG > IntM (Fig 1A).

RDFs have diverse roles in the control of MGE transfer. Several bacteriophage excisionases act as both RDFs and transcriptional regulators [35–44]. Phage-P2 Cox and the coliphage-186 Apl excisionases bind and bend *attP* and *attL* DNA to promote prophage excision, but they also stimulate induction of the lytic cycle by blocking transcription of repressor genes *cl* and *c*, respectively [35–43]. The Cox protein additionally stimulates derepression of neighbouring P4 prophages by activating transcription from the late P4-phage promoter [39, 45]. Cox-bound promoter and *attP* regions each contain six or more repeats of a “cox-box” consensus sequence that may vary in direction or percentage identity between different binding targets, and may be bound with variable affinity [38, 39, 42]. A protein sharing structural homology with excisionases has recently been shown to be essential for relaxasome processing of the conjugative plasmid pIP501 [46]. These examples and our findings here emphasise that RDFs/excisionases have evolved differential and evolutionarily flexible roles in the control of MGE dissemination.

The RdfS proteins of R7A and WSM1271 are almost identical at the amino-acid level apart from the extreme C-terminus (S1 Fig). Therefore, it is possible that the *rdfG* and *rdfM* promoter regions could have evolved DNA-binding targets that respond to RdfS, rather than RdfS having evolved specific new functions associated with ICE^3^_._ We were unable to identify any clearly conserved DNA sequence motifs on *attL*_*S*_, *attP*_*S*_ or the *rdfG* or *rdfM* promoter regions. However, excisionase binding sites are often poorly conserved at the DNA-sequence level and for most the mode of site recognition is not well understood. Most characterized RDFs have a winged-helix-turn-helix structure that contacts both major and minor DNA grooves, therefore overall DNA topology is believed to be especially critical for recognition [47]. Given that RdfS presumably binds multiple distinct sites on ICE*Mc*Sym^1271^, RdfS presents itself as an enticing research focus for gaining a deeper understanding of excisionase-DNA recognition characteristics and the multifaceted roles of excisionases in stimulating horizontal transfer of diverse MGE.

ICE*Mc*Sym^1271^-α carries two functional QS-sensor genes, *traR1* and *traR2*. Sequence comparisons of the ICE*Ml*Sym^R7A^ and ICE*Mc*Sym^1271^ QS loci suggest that the ICE*Mc*Sym^1271^-derived TraR2 protein is the more immediate orthologue of R7A-derived TraR. Broader comparisons of the QS loci organisation between these ICEs suggest that each ICE may have evolved from an ancestral ICE carrying two complete sets of *traR-traI* loci (S6 Fig). The DNA sequence upstream of *traI1* on ICE*Ml*Sym^R7A^ lacks a *traR1* homologue but does contain sequence homologous to the 3’-end of *traR1* from ICE*Mc*Sym^1271^, suggesting deletion of an ancestral copy of *traR1* has occurred in R7A. The *traI2* gene on ICE*Mc*Sym^1271^ appears to have become a pseudogene with several internal truncations, but a truncated seemingly nonsense open-reading-frame remains that has retained both its position relative to the upstream *tra* box and translational overlap with *msi172*, as is the case on other related ICEs (S5 Fig). On ICE*Ml*Sym^R7A^, *traI2* is a complete and potentially functional gene, but ICE*Ml*Sym^R7A^ excision or transfer is unaffected for a markerless deletion *traI2* mutant, suggesting it too may be in the early stages of pseudogenisation.

For both ICE*Mc*Sym^1271^ and ICE*Ml*Sym^R7A^ the functional AHL-synthase *traI1* and the apparent *traI2* pseudogene that is translationally coupled to *msi172-msi171* are proceeded by a *tra*-box sequence allowing for transcriptional control by TraR. ICE*Ml*Sym^R7A^ is exquisitely sensitive to overexpression of *msi172-msi171* or *rdfS*, which cause growth inhibition and loss of ICE*Ml*Sym^R7A^ respectively [23, 31, 48]. In the presence of AHLs, expression of *traI2-msi172-msi171* in R7A is lower than that observed for *traI1* [27]. Our RNAseq data similarly indicates that that expression from the ICE*Mc*Sym^1271^ *traI1* promoter is stronger than from the *traI2-msi172-msi171* promoter (Table 2, S4A and S4B Fig). As previously speculated [27], this separation of QS-activated genes involved in stimulation of excision (*msi172-msi171*) and AHL-production (*traI1*) has likely facilitated independent adjustment of expression levels from each QS-activated promoter. This type of genetic uncoupling of AHL synthase genes from other QS-activated genes could in some instances explain the presence of orphan–or solo—QS regulators and AHL-synthase genes frequently identified throughout gram-negative bacteria [49, 50].

ICE^3^s are a novel and unexpected form of MGE and the three-integrase system seemingly introduces considerable unnecessary complexity. However, in this work we show that the activity of RdfS as a master regulator of ICE^3^ excision greatly simplifies the pathway to excision. With RdfS in control, the excisive recombination reactions are induced in a predetermined order to excise ICE*Mc*Sym^1271^. As previously discussed [19, 26], despite the complex arrangement of integrase *att* sites, the formation of the prototype ICE^3^ may have occurred following only two chromosomal inversions between three single-part ICEs or non-conjugative integrating elements. We also suspect that the regulatory control of RdfS over *rdfG* and *rdfM* transcription could have pre-existed ICE^3^ on these ancestral single-part constituents. Several putative symbiosis ICEs carry *rdfS* but lack an associated IntS gene and instead carry a unique integrase and distinct *attL* site within one of five serine tRNA genes (*Mesorhizobium* spp. strains CC1192 [51]; WSM3873 (NZ_LYTM00000000.1), AA23 (NZ_LYTP00000000.1) and WSM3859 (NZ_NSGG00000000.1)). Moreover, numerous more distantly related putative ICEs in the α-proteobacteria carry a homologue of *rdfS* but lack an obvious *intS* homologue [28]. The conservation of *rdfS* but lack of conservation of *intS* on these ICEs suggests that RdfS homologues may be able stimulate excisive recombination through interactions with multiple distinct recombination systems. With this view in mind, the evolution of ICE^3^ and capture of unique ICE genes [26] potentially involves recombination between groups of distinct ICE^3^, ICEs and non-conjugative integrative elements that already share common regulatory control elements. In summary, this work provides substantial insight into the molecular control and evolution of these complex tripartite elements.

## Materials and methods

### Bacteria, plasmids, and growth conditions

Strains and plasmids are listed in Table 3. Strains were cultured as previously described [23, 27, 29, 52, 53]. Allelic replacement, and markerless deletion mutants were constructed using double crossover homologous recombination as previously described [23]. Plasmids for construction of mutants are described in Table 3 and primers used are listed in S1 Table. Construction of plasmids is detailed in Supplementary materials and methods (S1 File).

**Table 3:** Bacterial strains and plasmids.

Strain	^a^ Relevant Characteristics	Reference
***Escherichia coli*** DH10B	F^-^ *endA1* *deoR*^+^ *recA1* *galE15* *galK16* *nupG* *rpsL* Δ*(lac)X74* φ80*lacZΔM15 araD139* Δ*(ara*,*leu)7697 mcrA* Δ*(mrr-hsdRMS-mcrBC)* Str^R^ λ^–^	Invitrogen
ST18	S17 Δ*pir* Δ*hemA*	[54]
***Chromobacterium violaceum***		
CV026	Biosensor strain for detection of C_4_-C_8_ *N-*acyl-homoserine lactones	[29]
***Mesorhizobium ciceri***		
WSM1271	*Bisserula pelecinus* symbiont, harbours ICE*Mc*Sym^1271^ (accession NC_014923.1)	[55]
1271Δ*rdfG*::Ω*aadA*	WSM1271 *rdfG* Ω*aadA* replacement mutant	This study
1271Δ*rdfM*::Ω*aadA*	WSM1271 *rdfM* Ω*aadA* replacement mutant	This study
1271Δ*rdfS*	WSM1271 *rdfS* in frame deletion mutant	This study
***M*. *loti***		
R7ANS	Symbiosis ICE cured derivative of *M*. *loti* R7A	[23]
**Plasmids**		
pJQ200 SK	Suicide vector in *Mesorhizobium*, contains *sacB*, GmR	[56]
pEX18Tc	Suicide vector in *Mesorhizobium*, contains *sacB*, TcR	[57]
pHP45Ω	Insertional inactivation vector carrying an Ω*aadA1* cassette, SmR, SpR	[58]
pJET-*aadA*	pJET 1.2 carrying the Ω*aadA* cassette from pHP45Ω amplified using primers 34 & 35, SmR, SpR, ApR	This study
pJQΩ*rdfG*	pJQ200 SK carrying the Ω*aadA* cassette from pHP45Ω flanked by regions upstream and downstream of *rdfG* amplified using primers 1, 2 & 3, 4, respectively, used to create 1271Δ*rdfG*::Ω*aadA*, SmR, SpR GmR	This study
pJQΩ*rdfM*	pJQ200 SK carrying the Ω*aadA* cassette from pHP45Ω flanked by regions upstream and downstream of *rdfM* amplified using primers 5, 6 & 7, 8, respectively, used to create 1271Δ*rdfM*::Ω*aadA*, SmR, SpR GmR	This study
pEXΔ*rdfS*	pEX18Tc carrying regions flanking *intS* amplified using primers 9, 10 & 11, 12 respectively, used to create WSM1271Δ*rdfS*, TcR	This study
pJP2	Stable (contains Par region), low copy number BHR IncP vector, TcR	[59]
pJP2-*rdfG*	pJP2 carrying *rdfG* from WSM1271 amplified using primers 13 & 14, TcR	This study
pJP2-*rdfM*	pJP2 carrying *rdfM* from WSM1271 amplified using primers 15 & 16, TcR	This study
pJP2-*rdfS*	pJP2 carrying *rdfS* from WSM1271 amplified using primers 17 & 18, TcR	This study
pPR3	pPROBE-KT carrying the *nptII* promoter from pFAJ1708, NmR	[60–62]
pPR3-*rdfG*	pPR3 carrying *rdfG* from WSM1271 amplified using primers 19 & 20, NmR	This study
pPR3-*traI1*	pPR3 carrying *traI1* from WSM1271 amplified using primers 21 & 22, NmR	This study
pSacB	BHR vector carrying inducible IPTG promoter and *sacB* gene, NmR	[19]
pSacB-*rdfM*	pSacB carrying *rdfM* from WSM1271 amplified using primers 23 & 16, NmR	This study
pSDz	BHR plasmid, carries IPTG inducible promoter and promoterless *lacZ*, TcR	[31]
pSDz-*traR1*	pSDz carrying *tra1R* from WSM1271 amplified using primers 24 & 25, TcR	This study
pSDz-*traR2*	pSDz carrying *traR2* from WSM1271 amplified using primers 36 & 37, TcR	This study
pSDz-*msi172171*	pSDz carrying *msi172-msi171* from WSM1271 amplified using primers 26 & 27, TcR	This study
pSDz-P*rdfG*	pSDz carrying the *rdfG* promoter from WSM1271 amplified using primers 28 & 29, TcR	This study
pSDz-P*rdfM*	pSDz carrying the *rdfM* promoter from WSM1271 amplified using primers 30 & 31, TcR	This study
pSDz-P*rdfS*	pSDz carrying the *rdfS* promoter from WSM1271 amplified using primers 32 & 33, TcR	This study
pSDz*P*_*traI1*_*-lacZ*	pSDz carrying the *traI* promoter from WSM1271 amplified using primers 38 & 39, TcR	This study
pSDz*-traR1P*_*traI1*_*-lacZ*	pSDz-*traR1* carrying the *traI* promoter from WSM1271 amplified using primers 38 & 39, TcR	This study
pSDz-*traR2P*_*traI1*_*-lacZ*	pSDz-*traR2* carrying the *traI* promoter from WSM1271 amplified using primers 38 & 39, TcR	This study
pTHQP-1	Standard construct for qPCR assays for ICE^3^ excision, GmR	[19]
pJET 1.2.	Commercial blunt cloning vector, ApR	Thermo Fisher Scientific

^**a**^ Abbreviation for antibiotic resistances are as follows; ApR, ampicillin; GmR, gentamycin; NmR, neomycin; SpR, spectinomycin; SmR, streptomycin; TcR, tetracycline. See S1 Table for primer details.

### Molecular techniques, assays, and bioinformatics

DNA extractions, purifications, electrophoresis and PCR were carried out as previously described [18, 23]. Sanger sequencing was performed by the Australian Genome Research Facility. Nucleotide and amino acid alignments were performed using the T-Coffee multiple sequence aligner [63]. Protein secondary structures were predicted using Jpred(v4) [64]. Synteny comparisons were performed using the Artemis Comparison Tool [65] and plotted with genoplotR [66]. β-galactosidase assays were performed as previously described with three to six biological replicates per treatment [31, 67]. *Mesorhizobium* conjugation experiments were performed as previously described [19]. CV026 bioassays were performed on *E*. *coli* strains by streaking them adjacent to CV026 on LB agar and plates and incubating these plates for 24 h at 28°C [29]. CV026 well-diffusion bioassays were performed on *M*. *loti* strains as previously described [28, 29]. All cloning was carried out in *E*. *coli* DH10B and constructs were chemically transformed [68] into *E*. *coli* ST18 for mobilisation into *Mesorhizobium* spp. via biparental mating [54].

### qPCR assays for ICE^3^ excision

Genomic DNA for qPCR was extracted from 64-h TY broth cultures as previously described [19]. Our previously validated qPCR assay [19] was used to measure the percentage of chromosomes carrying each individual *attB* (*attB*_*G*,_ *attB*_*M*_, and *attB*_*S*_) and corresponding *attP* (*attP*_*G*,_ *attP*_*M*_, and *attP*_*S*_) site in samples of genomic DNA extracted from WSM1271 cultures. This was achieved by comparing the standardised relative abundance of each *attP* and *attB* site to the chromosomal gene *melR*. Primer sites for the qPCR assay are shown in Fig 1, and described in S1 Table.

### RNA-Sequencing and statistical analysis

TY broth cultures (OD_600_ 0.8–1.0) were grown for RNAseq experiments as previously described [69] with three biological repetitions per treatment and two technical repetitions per sample. Total RNA was isolated as previously described [70]. RNA quality and concentration was analysed at various points throughout processing using Experion StdSense or HighSens analysis kit assays (Bio-Rad Technologies). DNA was removed from approximately 3 μg of total RNA using the TURBO DNA-free kit (Invitrogen) and confirmed using a Qubit fluorometer dsDNA BR assay. rRNA was depleted from total RNA using a Ribo-Zero rRNA magnetic kit (Illumina) and resulting RNA was purified using a RNA Clean & Concentrator (Zymo Research). Barcoded cDNA libraries were prepared from rRNA depleted RNA samples using Ion Total RNA-Seq kit v2 (Thermo Fisher). Each barcoded cDNA library was diluted in DEPC treated milliQ water to a final concentration of 75 pM and templates for sequencing were prepared using an Ion Chef instrument (Thermo Fisher). Sequencing was performed using the Ion Proton system (Thermo Fisher). Read sets from technical repetitions were combined. Adapter sequences were removed using nesoni clip (http://www.vicbioinformatics.com/software.nesoni.shtml). To reduce any potential rRNA/total-RNA abundance biases introduced during rRNA depletion, reads mapping to rRNA genes were removed using FastQ Screen (https://www.bioinformatics.babraham.ac.uk). Reads were mapped to the WSM1271 genome (accession NC_014923) using Bowtie 2 [71] and visualised using Artemis [72] or Integrated Genome Browser [73]. For gene expression analysis, read sets were additionally filtered to remove sequences matching plasmids pPR3*-traI1* and pSDz*-traR1* prior to mapping. An average (per biological replicate) of 14 million (standard deviation (SD) = 3.3 million) QS+ and 8.5 million (SD = 1.5 million) QS- post-filter reads were mapped to WSM1271 with 96.7–98.6% alignment rate. Read counts for gene features were performed using HTSeq [74] with default settings then imported into DESeq2 [33] for identification of differentially expressed genes (S1 Dataset).

To measure expression from the *traI1* and *traI2* promoter regions, the unfiltered reads were mapped to the WSM1271 chromosome using the procedures described above, and read counting was performed using the—nonunique all function on HTSeq so that reads mapping ambiguously to the *traI1* and *traI2* regions and ORFs were counted for both features.

## Supporting information

S1 Fig Predicted secondary structures of RdfG, RdfM, and RdfS. Secondary structures were predicted using Jpred(v4) [64]. α-helices are highlighted in yellow, β-sheets are highlighted in blue. All three proteins carry a predicted two stranded MerR-family winged helix-turn-helix motif characteristic of RDFs [25].
(TIF)

S2 Fig Production of AHLs by TraI1. The *C*. *violaceum* CV026 biosensor strain [29] was used to detect the production of AHLs in (A) *E*. *coli* DH10B or (B) *M*. *loti* R7ANS either constitutively expressing ICE*Mc*Sym^1271^-derived *traI1* from the plasmid pPR3-*traI1*, or carry the empty vector pPR3. Production of a purple violacein halo indicated production of C_4_-C_8_ AHLs.
(TIF)

S3 Fig TraI1-dependent activation of the *traI1* promoter by TraR1 and TraR2. β-galactosidase assays [67] were performed on a set of R7ANS strains carrying the same *traI1* promoter-*lacZ* fusion on either pSDz, pSDz-*traR1*, or pSDz-*traR2*. These strains were induced for expression of *traR1/traR2* with 1 μM IPTG, and also carried either a constitutively expressed copy of *traI1* (pPR3-*traI1*), or the empty vector pPR3. Assays were performed with three biological replicates and mean β-galactosidase activity values (Relative Fluorescent Units/s/OD_600_) were compared by Bonferroni adjusted student’s t-tests. SD is denoted by error bars.
(TIF)

S4 Fig RNA-Seq mapping of the transcriptional start sites for *traI1*, *traI2*, and *rdfS*. The promoter regions of *traI1* (A), *traI2* (B), and *rdfS* genes (C) from WSM1271 were identified based on similarity with homologous regions in R7A. Nucleotide alignments were performed using the T-Coffee multiple sequence aligner [63]. Transcriptional start sites for R7A genes previously mapped by 5’RACE are shown in bold [27, 31]. Relative read depth (or sequencing depth) plots represent a standardised value for the mean number of reads mapped to the positive strand of the regions shown in this figure from the three unfiltered QS+ transcriptome libraries of WSM1271.These plots were produced using Integrated Genome Browser [73]. QS+ strains were induced for QS by overexpressing both *traI1* and *traR1* from the plasmids pPR3-*traI1* and pSDz*-traR1*, respectively. Mean values of 2196.16 ± (SD) 434.70 TPM unfiltered reads and 660.88 ± 276.84 TPM unfiltered reads were mapped to the non-coding regions between the transcriptional start sites and start codons for *traI1* and *traI2*, respectively. A students t-test revealed that this difference was significant (*P =* 0.01).
(TIF)

S5 Fig Alignment of *traI2* promoter regions and TraI2 protein sequences in diverse *Mesorhizobium* spp. (A) The nucleotide sequence of *traI2* promoters and (B) the TraI2 amino acid sequences from six *Mesorhizobium* strains were aligned using the T-coffee multiple sequence aligner [63].
(TIF)

S6 Fig Possible evolution of QS loci on ICE*Ml*Sym^R7A^ and ICE*Mc*Sym^1271^. On ICE*Ml*Sym^R7A^, *traR* is encoded upstream of an operon encoding the likely non-functional AHL-synthase gene *traI2*, *msi172-msi171* and *qseM-qseC*. The functional AHL synthase TraI1 is encoded at a separate location. ICE*Mc*Sym^1271^ carries *traR2* upstream of *qseM*-*qseC*, however, the *traI2-msi172-msi171* region has been translocated to a different position and *traI2* has become internally truncated. ICE*Ml*Sym^1271^ carries a second *traR* gene *traR1* paired with the *traI1* gene. It is likely that ICE*Ml*Sym^R7A^ originally had a *traR1* gene that has subsequently been deleted. Consistent with this notion, the 100-bp upstream of *traI1* closely resembles the 3’-end of *traR1*. Thus, it seems likely that an ancestral ICE carried an operon comprising *traR2-traI2-msi172-msi171* upstream of divergent *qseC* and *qseM* genes and a second QS locus containing *traR1-traI1*. Synteny comparisons were performed using the Artemis Comparison Tool [65] and plotted with genoplotR [66].
(TIF)

S1 Table Oligonucleotides used in this study. (PDF)

S1 Dataset TPM values and DESeq2 output for differential gene expression analysis. (XLSX)

S1 File Supplementary materials and methods. (DOCX)
