# Supplementary figures and images for: Sequential induction of three recombination directionality factors directs assembly of tripartite integrative and conjugative elements
Source: PLoS Genet. 2018 Mar 22;14(3):e1007292. doi: 10.1371/journal.pgen.1007292 (PMC5882170; doi:10.1371/journal.pgen.1007292)

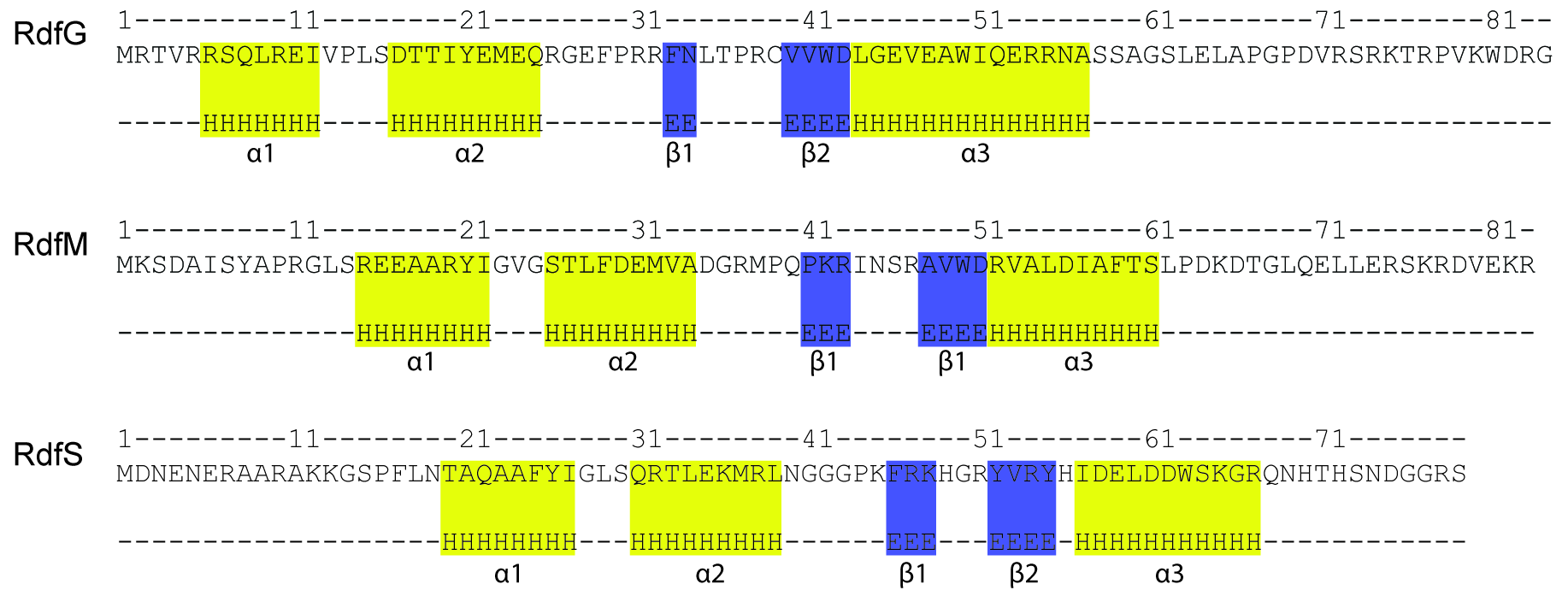

Supplement: S1 Fig — Secondary structures were predicted using Jpred(v4) [64]. α-helices are highlighted in yellow, β-sheets are highlighted in blue. All three proteins carry a predicted two stranded MerR-family winged helix-turn-helix motif characteristic of RDFs [25]. (TIF) [file pgen.1007292.s001.tif]

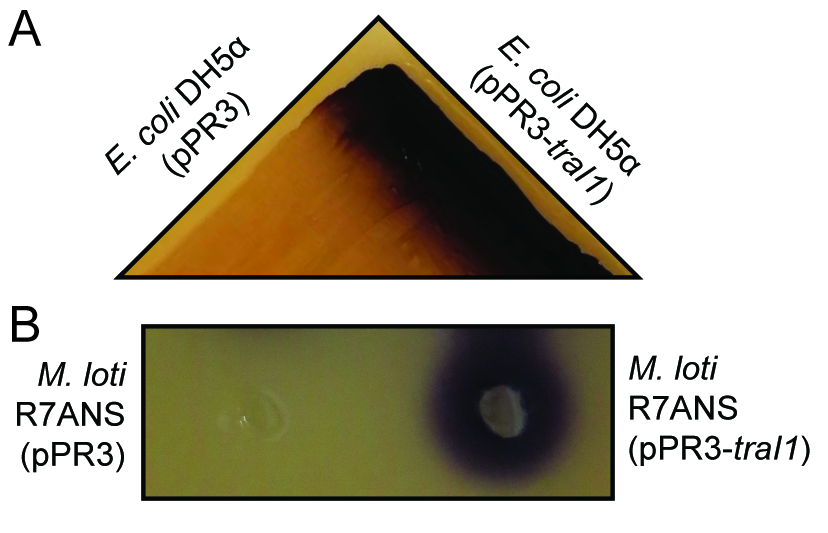

Supplement: S2 Fig — The C. violaceum CV026 biosensor strain [29] was used to detect the production of AHLs in (A) E. coli DH10B or (B) M. loti R7ANS either constitutively expressing ICEMcSym1271-derived traI1 from the plasmid pPR3-traI1, or carry the empty vector pPR3. Production of a purple violacein halo indicated production of C4-C8 AHLs. (TIF) [file pgen.1007292.s002.tif]

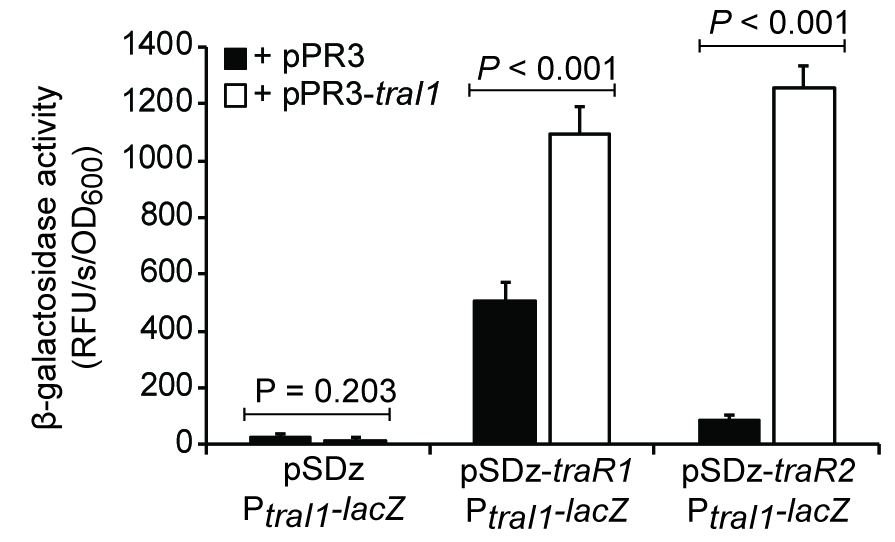

Supplement: S3 Fig — β-galactosidase assays [67] were performed on a set of R7ANS strains carrying the same traI1 promoter-lacZ fusion on either pSDz, pSDz-traR1, or pSDz-traR2. These strains were induced for expression of traR1/traR2 with 1 μM IPTG, and also carried either a constitutively expressed copy of traI1 (pPR3-traI1), or the empty vector pPR3. Assays were performed with three biological replicates and mean β-galactosidase activity values (Relative Fluorescent Units/s/OD600) were compared by Bonferroni adjusted student’s t-tests. SD is denoted by error bars. (TIF) [file pgen.1007292.s003.tif]

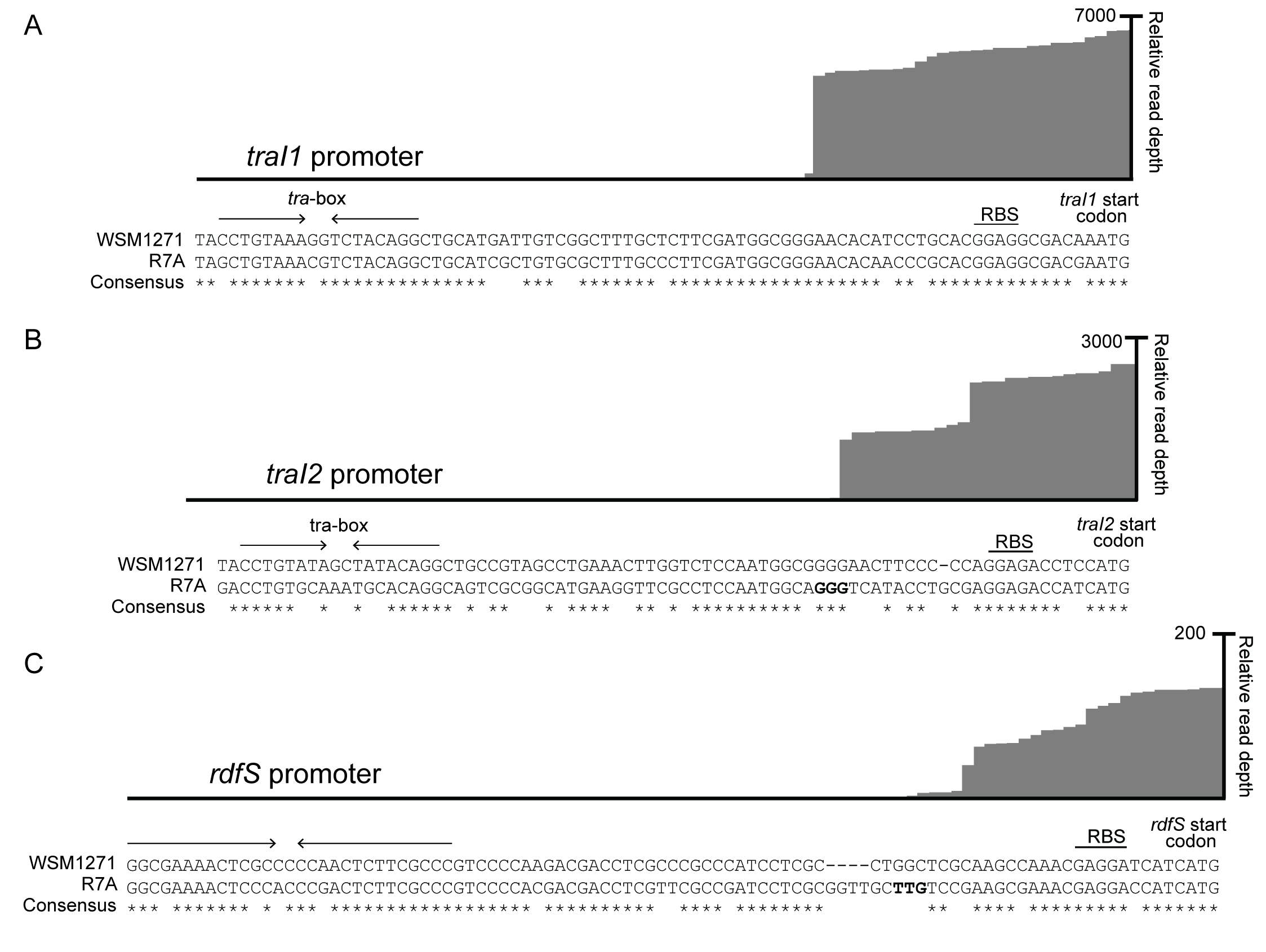

Supplement: S4 Fig — The promoter regions of traI1 (A), traI2 (B), and rdfS genes (C) from WSM1271 were identified based on similarity with homologous regions in R7A. Nucleotide alignments were performed using the T-Coffee multiple sequence aligner [63]. Transcriptional start sites for R7A genes previously mapped by 5’RACE are shown in bold [27, 31]. Relative read depth (or sequencing depth) plots represent a standardised value for the mean number of reads mapped to the positive strand of the regions shown in this figure from the three unfiltered QS+ transcriptome libraries of WSM1271.These plots were produced using Integrated Genome Browser [73]. QS+ strains were induced for QS by overexpressing both traI1 and traR1 from the plasmids pPR3-traI1 and pSDz-traR1, respectively. Mean values of 2196.16 ± (SD) 434.70 TPM unfiltered reads and 660.88 ± 276.84 TPM unfiltered reads were mapped to the non-coding regions between the transcriptional start sites and start codons for traI1 and traI2, respectively. A students t-test revealed that this difference was significant (P = 0.01). (TIF) [file pgen.1007292.s004.tif]

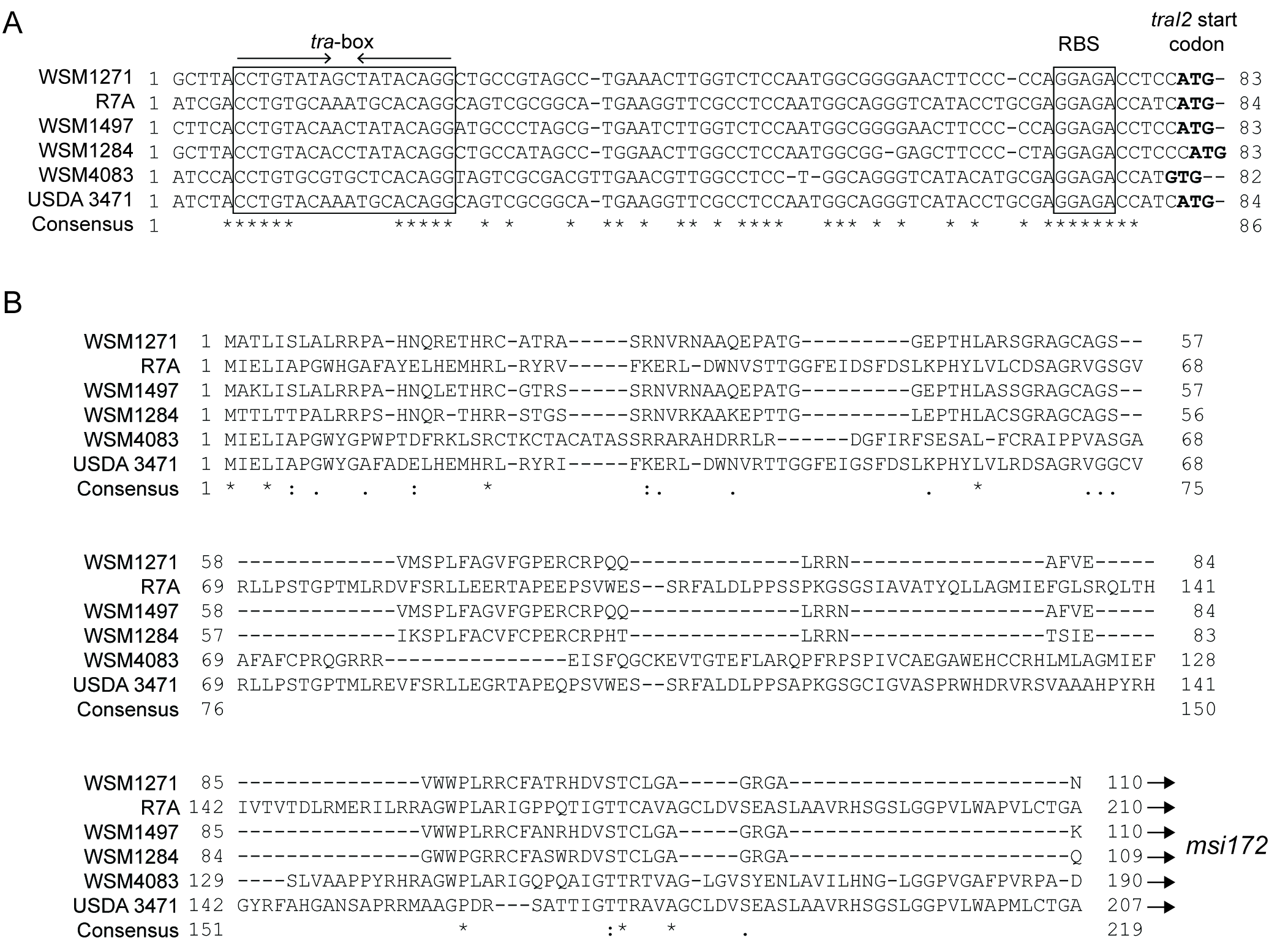

Supplement: S5 Fig — (A) The nucleotide sequence of traI2 promoters and (B) the TraI2 amino acid sequences from six Mesorhizobium strains were aligned using the T-coffee multiple sequence aligner [63]. (TIF) [file pgen.1007292.s005.tif]

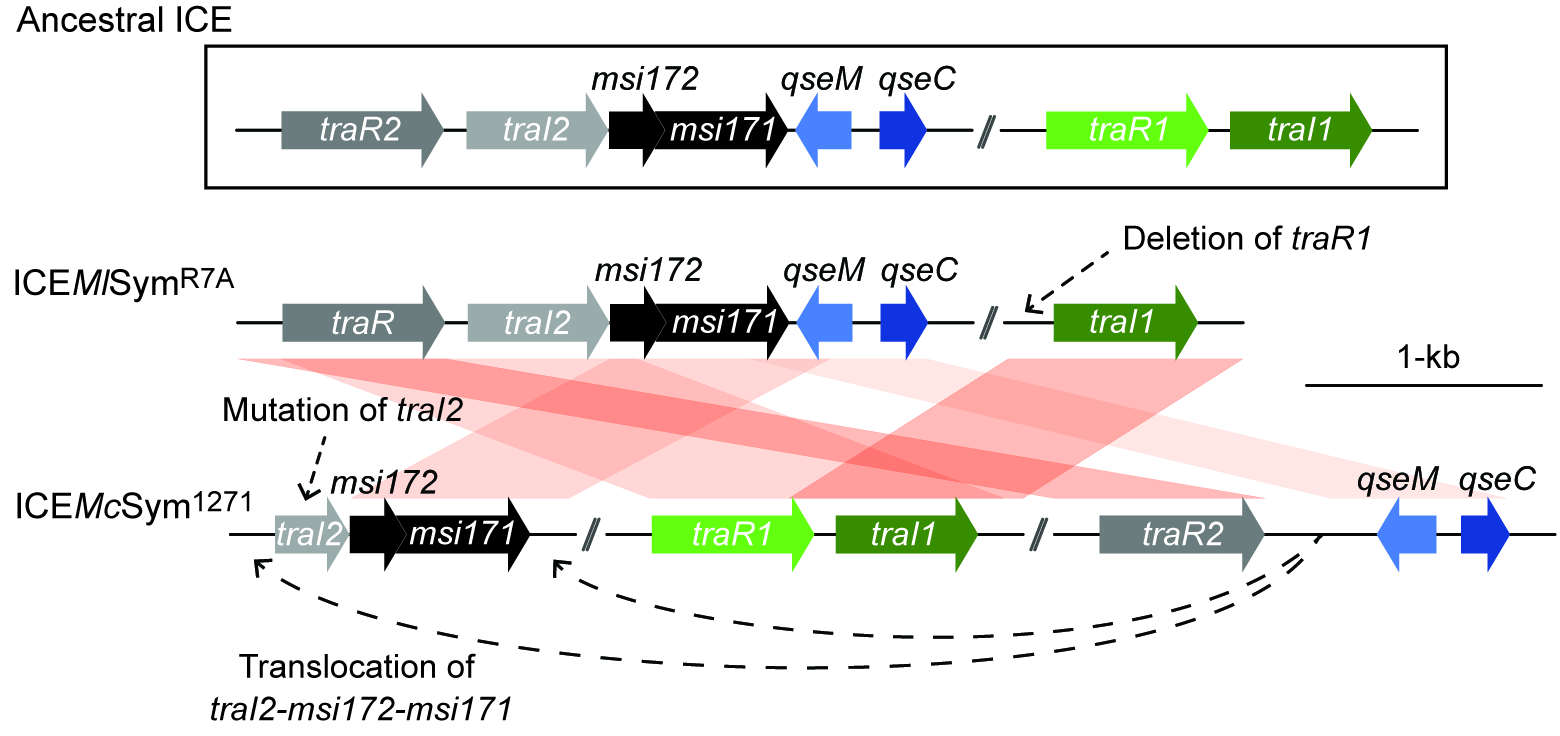

Supplement: S6 Fig — On ICEMlSymR7A, traR is encoded upstream of an operon encoding the likely non-functional AHL-synthase gene traI2, msi172-msi171 and qseM-qseC. The functional AHL synthase TraI1 is encoded at a separate location. ICEMcSym1271 carries traR2 upstream of qseM-qseC, however, the traI2-msi172-msi171 region has been translocated to a different position and traI2 has become internally truncated. ICEMlSym1271 carries a second traR gene traR1 paired with the traI1 gene. It is likely that ICEMlSymR7A originally had a traR1 gene that has subsequently been deleted. Consistent with this notion, the 100-bp upstream of traI1 closely resembles the 3’-end of traR1. Thus, it seems likely that an ancestral ICE carried an operon comprising traR2-traI2-msi172-msi171 upstream of divergent qseC and qseM genes and a second QS locus containing traR1-traI1. Synteny comparisons were performed using the Artemis Comparison Tool [65] and plotted with genoplotR [66]. (TIF) [file pgen.1007292.s006.tif]
