# Supplementary material for: Sequential induction of three recombination directionality factors directs assembly of tripartite integrative and conjugative elements
Source: PLoS Genet. 2018 Mar 22;14(3):e1007292. doi: 10.1371/journal.pgen.1007292 (PMC5882170; doi:10.1371/journal.pgen.1007292)
Supplement: S1 Table — (PDF) [file pgen.1007292.s007.pdf]

| # | Primer | [a]Sequence | Source |
|---|---|---|---|
| **Cloning primers** | | | |
| 1 | Ω*rdfG*_Up_5'_SacI | ATCAG**GAGCTC**AAGCAGCGTGACAAGCGGC | This study |
| 2 | Ω*rdfG*_Up_3'_XhoI | ATCAG**CTCGAG**GTCAAATGGGATCGAGGATGACGG | This study |
| 3 | Ω*rdfG*_Dn_5'_XbaI | ATCAG**TCTAGA**AATCCGTCGCGCCTCAATGT | This study |
| 4 | Ω*rdfG*_Dn_3'_NotI | ATCAG**GCGGCC**GCTTGCCCGGCTGGGCCTT | This study |
| 5 | Ω*rdfM*_Up_5'_SacI | ATCAG**GAGCTC**CCACGCAAGCGCAGCG | This study |
| 6 | Ω*rdfM*_Up_3'_XhoI | ATCAG**CTCGAG**ACGCTTGTTGCGTATACGCTGTAGAC | This study |
| 7 | Ω*rdfM*_Dn_5'_XbaI | ATCAG**TCTAGA**GGACGCTGCCTCGGTCCT | This study |
| 8 | Ω*rdfM*_Dn_3'_NotI | ATCAG**GCGGCCGC**GTCACCTGTCAACGATCGGCAAG | This study |
| 9 | ∆*rdfS*_Up_5'_gib | **ACTAAAGGGAACAAAAGCTGGAGCT**CGGCATCGTACCCCGGTCG | This study |
| 10 | ∆*rdfS*_Up_3'_gib | **TGGGTGTGGTTCT**CCTTTTTGGCGCGGGCGG | This study |
| 11 | ∆*rdfS*_Dn_5'_gib | **CGCGCCAAAAAGG**AGAACCACACCCATTCCAACGATG | This study |
| 12 | ∆*rdfS*_Dn_3'_gib | **TTGGGTACCGGGCCCCCCCTCGAGG**TAGCGCTCGGGTCCGGCG | This study |
| 13 | *rdfG*_5'_HindIII | ATCAG**AAGCTT**GTTCGCCGTCCGCTCAATC | This study |
| 14 | *rdfG*_3'_XbaI | ATCAG**TCTAGA**TCATCCTCGATCCCATTTGACG | This study |
| 15 | *rdfM*_5'_HindIII | ATCAG**AAGCTT**AGCAAGCCTATTCTGGTGGCCG | This study |
| 16 | *rdfM*_3'_XbaI | ATCAG**TCTAGA**TTATCGTTTTTCAACGTCCCGTTTGCT | This study |
| 17 | *rdfS*_5'_HindIII | ATCAG**AAGCTT**GCCGAGGAGCGGCGAAA | This study |
| 18 | *rdfS*_3'_XbaI | ATCAG**TCTAGA**TCATGAGCGGCCTCCATCGT | This study |
| 19 | *rdfG*_5'_BamHI | ATGAC**GGATCC**ACATTGAGGCGCGACGGATT | This study |
| 20 | *rdfG*_3'_KpnI | ATGAC**GGTACC**TCATCCTCGATCCCATTTGACG | This study |
| 21 | *traI1*_5'_KpnI | ATCTA**GGTACC**GGAGGCGACGAATGATGCAGCTAATCACACCTGAGC | This study |
| 22 | *traI1*_3'_KpnI | ATCTA**GGTACC**TTAAGCGTATGCCGGCAGGC | This study |
| 23 | *rdfM*_5'_SacI | ATCAG**GAGCTC**GGAGGCGACGAATGAAGAGTGACGCAATCTCGTATGCC | This study |
| 24 | *traR*_5'_PstI | ATCTA**CTGCAG**GGAGGCGACGAATGCATCGCGTGTTTGAAAATTTC | This study |
| 25 | *traR*_3'_XbaI | ATCTA**TCTAGA**TCAGGATCTCGAATGTCGGGAA | This study |
| 26 | *msi172*_5'_gib | **TAACAATTTCACACATAGCTAACTG**GGAGGCGACGAATGCCTGCAGTTCT CGTG | This study |
| 27 | *msi171*_3'_gib | **CTTTAGATGCCGCTTCTTTTGCAGA**TCAAAGAAGGAAATCCCTGTACCC | This study |
| 28 | P*rdfG*_5'_XhoI | ATGAC**CTCGAG**TGCTCGTGAGCAAGACCTAGGCTT | This study |
| 29 | P*rdfG*_3'_XhoI | ATGAC**CTCGAG**AATCCGTCGCGCCTCAATGT | This study |
| 30 | P*rdfM*_5'_XhoI | ATGAC**CTCGAG**TGGGTCGTTGATCGCCAGC | This study |
| 31 | P*rdfM*_3'_XhoI | ATGAC**CTCGAG**GGACGCTGCCTCGGTCCT | This study |
| 32 | P*rdfS*_5'_XhoI | ATGAC**CTCGAG**TCCGGCCGACCCGAG | This study |
| 33 | P*rdfS*_3'_XhoI | ATGAC**CTCGAG**GATGATCCTCGTTTGGCTTGCG | This study |
| 34 | *aadA1*_5'_Blunt | **ATGCATGTCGAC**GGAGCTGCATGTGTCAGAGGT | This study |
| 35 | *aadA1*_3'_Blunt | **GAGCTCGGTACC**GAGGCCCTTTCGTCTTCAAGA | This study |
| 36 | *traR2*_5'_SpeI | ATCAG**ACTAGT**GGAGGCGACGAATGACGAGGGACATGCCACTTGT | This study |
| 37 | *traR2*_3'_XbaI | ATCAG**TCTAGA**TCAGAGGATCGAGCTCCCTTGG | This study |
| 38 | P*traI1*_5'_BglII | ATCTA**AGATCT**CGACATTCGAGATCCTGATTCCTT | This study |
| 39 | P*traI1*_3'_XhoI | ATCTA**CTCGAG**TTGTCGCCTCCGTGCAGG | This study |
| **[b]QPCR primers** | | | |
| 40 | 1271attB(G)F | GCATCAACCGCGTCGTCTA | [19] |

| # | Primer | [a]Sequence | Source |
|---|---|---|---|
| 41 | 1271attB(G)R | GAAGTCTCCGGCAGCGAAA | [19] |
| 42 | 1271attB(M)F | GCTCCAGGTGTGCGTTTCT | [19] |
| 43 | 1271attB(M)R | TGGGTTGATTTGGGCGATCT | [19] |
| 44 | 1271attB(S)F | TGTCTTTGGGCTTAGCGTTCT | [19] |
| 45 | 1271attB(S)R | ACAGGCCCAGATAGCTCAGTT | [19] |
| 46 | ICE*Mc*Sym1271(G)F | CAGTCTGCAGCAACGATGAC | [19] |
| 47 | ICE*Mc*Sym1271(G)R | CAGTGTGTTGAAATTCCGGTTGA | [19] |
| 48 | ICE*Mc*Sym1271(M)F | GACCGTGGTCTTTGCTTTGG | [19] |
| 49 | ICE*Mc*Sym1271(M)R | TCTCCGAACGTCCGCAAA | [19] |
| 50 | ICE*Mc*Sym1271(S)F | GGAACCGAACCAATCCACAGA | [19] |
| 51 | ICE*Mc*Sym1271(S)R | TGCCGAAACAGAAGCGTAGA | [19] |
| 52 | 1271melRF | CTGATGTCACCAGTGTTGCG | [19] |
| 53 | 1271melRR | CGCCCAGGTCGAGGTTAATT | [19] |

[a] Text in bold demarks a restriction site (detailed in the primer name), or overlapping region for Gibson cloning. Underlined text demarks an artificially introduced RBS.

[b] QPCR primer targets sites are as follows; $attB_G$ 44 & 45; $attB_M$ 46 & 47; $attB_S$ 48 & 49; $attP_G$ 50 & 51; $attP_M$ 52 & 53; $attP_S$ 54 & 55; *melR* 56 & 57.
