# Supplementary material for: Sequential induction of three recombination directionality factors directs assembly of tripartite integrative and conjugative elements
Source: PLoS Genet. 2018 Mar 22;14(3):e1007292. doi: 10.1371/journal.pgen.1007292 (PMC5882170; doi:10.1371/journal.pgen.1007292)
Supplement: S1 File — (DOCX) [file pgen.1007292.s009.docx]

# Supplementary information

## Primers and plasmid construction.

Primers used to construct plasmids in study are detailed in Table 3 and S1 Table. Plasmids were constructed as follows;

**pJET-*aadA*.** The *aad*A gene was amplified by PCR from pHP45Ω and ligated as a blunt fragment into the commercial linearized cloning vector pJET 2.1.

**pEX∆*rdfS.*** Regions upstream and downstream of the *rdfS* gene were amplified by PCR from WSM1271 DNA and cloned into SalI/BamHI-digested pEX18Tc using Gibson assembly.

**pJQΩ*rdfG* and pJQΩ*rdfM.*** Regions upstream and downstream of *rdfG* and *rdfM* genes were amplified by PCR from WSM1271 DNA. Upstream fragments were digested with SacI/XhoI, downstream fragments were digested with XbaI/NotI, and the pJET*aadA* plasmid was digested with XhoI/XbaI to release the Ω*aadA* cassette. These three products were ligated with SacI/NotI digested pJQ200 SK and plated onto LB agar supplemented with gentamycin to select for the pJQ200 SK backbone, and spectinomycin to select for Ω*aadA*. The unique arrangement of restriction sites ensured that the final constructs comprised the pJQ200 SK backbone carrying the Ω*aadA* cassette flanked by the upstream and downstream regions of *rdfG* or *rdfM.*

**pJP2-*rdfG,* pJP2-*rdfM*, and pJP2-*rdfS*.** The *rdfG, rdfM,* and *rdfS* genes and upstream intergenic regions were amplified by PCR from WSM1271 DNA and cloned into pJP2 as HindIII-XbaI fragments.

**pPR3-*rdfG.*** The *rdfG* gene and its ribosome binding site (RBS) were amplified by PCR from WSM1271 DNA and cloned into pPR3 downstream of the *nptII* promoter as a BamHI-KpnI fragment.

**pPR3-*traI1.*** The *traI1* gene and an artificially introduced RBS were amplified by PCR from WSM1271 DNA and cloned into pPR3 downstream of the *nptII* promoter as a KpnI fragment.

**pSacB-*rdfM*.** The *rdfM* gene and an artificially introduced RBS were amplified by PCR from WSM1271 genomic DNA and cloned into pSacB downstream of the *lac* promoter as a XbaI-SacI fragment.

**pSDz-*traR1* and pSDz-*traR2.*** The *traR1* and *traR2* genes and an artificially introduced RBS were each amplified by PCR from WSM1271 DNA and cloned into pSDz downstream of the *lac* promoter as PstI-XbaI and SpeI-XbaI fragments respectively.

**pSDz-*msi172171.*** The *msi172-msi171* ORFs and an artificially introduced RBS were amplified by PCR from WSM1271 genomic DNA and cloned downstream of the *lac* promoter of EcoRI/HindIII-digested pSDz using Gibson assembly.

**pSDz-P*rdfG,* pSDz-P*rdfM*, and pSDz-P*rdfS*.** Non-coding regions upstream of the *rdfG, rdfM,* and *rdfS* genes (presumably capturing the native promoters) were amplified by PCR from WSM1271 DNA and cloned into pSDz downstream of the IPTG inducible promoter as XhoI fragments.

**pSDz*P_traI1_-lacZ,* pSDz-*traR1P_traI1_-lacZ,* and *pSDz-traR2P_traI1_-lacZ.*** The non-coding regions upstream of *traI1* was amplified by PCR from WSM1271 DNA and cloned into pSDz, pSDz-*traR1,* and pSDz-*traR* downstream of the promoterless *lacZ* genes as XhoI-BglII fragments.
